# Epidemiological and clinical evaluation of patients with a cleft in lower saxony Germany: a mono-center analysis

**DOI:** 10.1007/s00784-023-05187-9

**Published:** 2023-08-05

**Authors:** Kauffmann Philipp, Quast Anja, Schminke Boris, Kolle Johanna, Wolfer Susanne, Stepniewski Adam, Meyer-Marcotty Philipp, Schliephake Henning

**Affiliations:** 1 Department of Oral and Maxillofacial Surgery, University Medical Center Göttingen, Georg-August-University Göttingen, Robert-Koch-Straße 40, D-37099 Göttingen, Germany; 2 Department of Orthodontics, University Medical Center Göttingen, Göttingen, Germany; 3 Trauma Surgery, Orthopedics and Plastic Surgery, University Medical Center of Göttingen, Göttingen, Germany

**Keywords:** Cleft, Comorbidity, Treatment concept, Corrective surgery

## Abstract

**Objective:**

The aim was to provide epidemiological and clinical data on patients with orofacial clefts in Lower Saxony in Germany.

**Materials and methods:**

The records of 404 patients with orofacial clefts treated surgically at the University Medical Center Goettingen from 2001 to 2019 were analyzed in this retrospective study. Prevalence of orofacial clefts in general, orofacial clefts as manifestation of a syndrome, sex distribution, and prevalence of different cleft types was evaluated and associated with the need for corrective surgery, family history, pregnancy complications, and comorbidities.

**Results:**

The prevalence of orofacial clefts for Goettingen in Lower Saxony was 1:890. 231 patients were male and 173 were female. CLP was most common (39.1%) followed by CP (34.7%), CL (14.4%), CLA (9.9%), and facial clefts (2%). The left side was more frequently affected and unilateral cleft forms occurred more often than bilateral ones. Almost 10% of the population displayed syndromic CL/P. 10.9% of all patients had a positive family history regarding CL/P, predominantly from the maternal side. Pregnancy abnormalities were found in 11.4%, most often in the form of preterm birth. Comorbidities, especially of the cardiovascular system, were found in 30.2% of the sample. 2.2% of patients treated according to the University Medical Center Goettingen protocol corrective surgery was performed in form of a velopharyngoplasty or residual hole closure.

**Conclusions:**

The epidemiological and clinical profile of the study population resembled the expected distributions in Western populations. The large number of syndromic CL/P and associated comorbidities supports the need for specialized cleft centers and interdisciplinary cleft care.

## Introduction

Cleft lip and/or palate (CL/P) are not only among the most common orofacial deformities but are also among the most common congenital malformations [1, 2]. The prevalence of CL/P varies from 1 in 500 to 1 in 2500 births depending on sex, socioeconomic status, and geographic location [3, 4]. In European countries, a prevalence of 1:1000 is assumed [2]. However, no data for individual regions, especially Germany, exist. Furthermore, diverse phenotypes of CL/P occur at different frequencies, and complete CLP is found in 40–50% of patients, while CL±A and CP account for 20–25% and 30–35%, respectively. In the case of a unilateral cleft, the left side is more often affected than the right side [5]. Girls are generally less affected than boys by cleft lip with or without cleft palate [1, 6], while some authors assume that they are at higher risk of cleft palate only [7].

Cleft formation is proposed to be multifactorial, involving genetic and environmental factors, and a positive family history of CL/P increases the risk of cleft manifestation with different degrees of recurrence based on the severity and type of cleft [8, 9]. In addition to heredity, maternal exposure to drugs, infections, or poor nutrition affect the occurrence of CL/P and may contribute to one-third of CL/P cases [4, 10]. For example, a well-known exogenous factor that increases the risk for CL/P is maternal smoking, which leads to a 1.5 times higher likelihood of having offspring with CL/P. Further external factors include vitamin deficiencies (folic acid and vitamin B), oxygen deficiency, hypervitaminosis (vitamin A), viral infections, medications (e.g.: cortisone, anticonvulsants, and cytostatics), smoking, drugs, ionizing radiation, chemicals, alcohol, and stress [11–15].

Approximately 30% of CL/P cases are associated with congenital malformations and syndromes. Frequent birth defects accompanying CL/P include musculoskeletal, cardiovascular, and central nervous system defects [2, 16].

These numbers illustrate the complex epidemiological and clinical profiles of CL/P and emphasize the need for good interdisciplinary cooperation and the importance of specialized cleft centers [17, 18]. Thus, at the University Medical Center Goettingen, the cleft team consists of experts in the fields of cranio-maxillofacial surgery, orthodontics, otolaryngology, speech therapy, human genetics, prosthodontics, and conservative dentistry. In some cases, corrective surgery, i.e., lip revision, fistula closure, palate re-repair, and pharyngeal surgery, becomes necessary. However, corrective surgery is regarded as a burden on the patients and their families and should be avoided whenever possible. The likelihood of performing corrective surgery varies widely among different cleft centers, and the occurrence of lip revision ranges from 5% to 60% [19]. This could be due to different thresholds for corrective surgery but may also be based on the applied concepts in primary cleft care. In total, there are 201 cleft centers in Europe with 194 different treatment concepts[20, 21], and information on the rate of corrective surgeries by single centers is missing.

To date, there are few studies on the epidemiological and clinical profiles of individual regions in Europe, especially Germany. Therefore, the aim of the present study was to provide a comprehensive epidemiological and postoperative clinical overview of cleft care in the geographic area of Southern Lower Saxony. As the characterization of patients with clefts is essential for professionals involved in cleft care and for health services, we analyzed the patient collective of a single center with regard to their sex, clinical manifestation of the cleft, family and pregnancy history, comorbidities, and need for corrective surgery.

## Methods

This observational study was approved by the Ethics Committee of the University Medical Center Goettingen (21/4/20) in accordance with the Declaration of Helsinki. In a retrospective evaluation from 2001 to 2019, the records of all patients with a cleft who received surgery at the University Medical Center Goettingen were analyzed. Each patient was treated according to a consistent protocol (for details, see Table 1) and was seen once a year for follow-up. In this appointment, the current treatment needs were assessed and discussed with the patients and their parents. This cleft consultation was held once a month with the interdisciplinary team.

**Table 1:** Goettingen treatment protocol for patients with orofacial clefts

24–48 h after birth	Consultation of a cranio-maxillofacial surgeon and an orthodontist; manufacturing of a palatal plate to separate the oral and nasal cavities
From the 3rd month of age	Phoniatric examination, pedaudiological examination, and hearing test
5th–6th month of age	Lip closure at 5000–6000 g body weight and 10 g/dl Hb. If necessary, insertion of tympanostomy tubes In cases of facial cleft: cleft closure
12th month of age	Hard palate closure
15th–18th month of age	Soft palate closure In isolated cleft palate cases, closure is often performed earlier and simultaneously with hard palate closure
With the beginning of speech	If necessary, speech therapy
With onset of malocclusion from the age of 4 years	If necessary, early orthodontic treatment in primary/early mixed dentition to promote sagittal and transversal growth of the upper jaw
5–6 years of age	If necessary, columella lengthening and scar correction If necessary, velopharyngoplasty
8–11 years of age	If necessary, secondary osteoplasty
From the age of 8	If necessary, orthodontic treatment in the late mixed/permanent dentition to promote growth of the upper jaw and to adjust occlusion
15–18 years of age	If necessary, rhinoplasty, correction of scars
From the age of 21	If necessary, orthognathic surgery, insertion of implants

A total of 466 patients visited the cleft consultation center within the study period. The inclusion criteria were all patients with an orafacial cleft formation; the exclusion criterion was the absence of such a malformation. After record screening, many patients presented at the consultation with the suspicion of a cleft, which was not confirmed and was therefore not included in the analysis. After record screening, 404 patients were included as real patients with orofacial clefts in the analysis.

Written file documentation was evaluated by one person and spot-checked by a second person. The written records were systematically evaluated, and their sex (male, female), cleft type (CL, CLA, CLP, CP, facial cleft), sidedness (left, right), laterality (unilateral, bilateral), family and pregnancy history, comorbidities, the presence of a syndrome, and the number of surgeries, including corrective surgeries, were documented in a database using MS Access (Microsoft, Washington, USA). The determination of the cleft localization occurred at the time of the initial clinical presentation and documentation. The clarification of the syndrome was determined according to the documentation of the case, where all physician’s reports or treatment reports of the records were reviewed. The abnormalities during pregnancy and the term premature birth were determined by interviewing the parents or accompanying relatives.

Secondary surgeries were interventions on previously operated cleft regions. Here, a distinction must be made between interventions planned as part of the overall surgical concept (e.g., nasal Columella lengthening in patients with previously operated bilateral cleft lips and palates and secondary bone grafting) and corrective operations to improve the aesthetic and functional results in the case of unsatisfactory results of primary surgery.

### Statistics

The prevalence of clefts for the area of Goettingen in Lower Saxony was calculated as follows: $\frac{\textrm{Cleft}\ \textrm{patients}\ \textrm{born}\ \textrm{in}\ \textrm{Goettingen}\ \textrm{at}\ \textrm{the}\ \textrm{University}\ \textrm{Medical}\ \textrm{Center}\ \textrm{Goetttingen}\ }{\textrm{Live}\ \textrm{births}\ \textrm{in}\ \textrm{the}\ \textrm{city}\ \textrm{of}\ \textrm{Goettingen}}$. Since it can be assumed that all Patients with clefts born in Goettingen visited the University Medical Center Goettingen to be treated by the specialized cleft team, the number of live births in the city of Goettingen in the period from 2001 to 2019 was considered a reference value. The number of births in the city of Goettingen was obtained from the resident authority.

GraphPad Prism 5.0 (California, USA) was used to graphically display the data. Statistical testing was performed using SPSS (v.27, IBM, New York, USA). A one sample chi-squared test was used to determine whether sex, cleft type, and sidedness were equally distributed in the sample. To detect associations between the cleft type (CL vs. CLA vs. CLP vs. CP) and the frequencies of pregnancy abnormalities, syndromes, comorbidities, and the number of corrective surgeries, a chi-square test of independence was performed. When the expected frequencies were lower than 5, Fisher’s exact test was used. The level of significance was set at *α* = 0.05.

## Results

In total, 404 patients were included in the analysis. This corresponded to a prevalence of 1 in 890 births. A total of 231 patients (57.2%) were male, and 173 were female (42.8%), which means that the sex distribution had a ratio of 1.3:1 and differed significantly from the usual sex distribution of 1:1 in the general population (*p* = 0.005).

### Clinical manifestation of clefts

A total of 353 CLP patients were treated according to protocol. Forty-three (10.6% of 404) CLP patients had been treated elsewhere/outside of the protocol initially. Eight patients were treated due to a facial cleft and were not included in the classical CLP concept.

The occurrence of the 4 cleft types (CL, CLA, CLP, and CP) was not equally distributed in the study sample (*p*<0.001). A total of 8 (2%) patients had a facial cleft. In 58 (14.4%) patients, a CL was found, with a slightly higher prevalence in boys (*n*=32, 55.2%) than in girls (*n*=26, 44.8%; *p* = 0.512). The left side was more affected than the right side (unilateral left: *n*=34, 58.6%; unilateral right: *n*=15, 25.9%; *p*=0.009). A total of 7 (12%) patients had a bilateral CL. In 2 patients, the cleft side could not be determined from their records.

Forty (9.9%) patients had CLA, of which 18 (45%) were male and 22 (55%) were female (*p* = 0.636). Regarding laterality, 20 (50%) patients had a left-sided cleft, and 11 (27.5%) patients had a right-sided cleft (*p* = 0.15). In 8 (20%) patients, CLA was bilateral. In 1 patient, the cleft side was not documented.

A total of 158 (39.1%) patients were affected by CLP, of which 104 (65.8%) were male and 54 (34.2%) were female (*p* < 0.001). The analysis of sidedness revealed that CLP was more often left-sided (*n*=67, 42.4%) than right-sided (*n*=35, 22.2%; *p*=0.002). Bilateral CLP was found in 52 patients (32.9%). In 4 patients, the affected side could not be determined.

Patients with a CP were present in 140 (34.7%) cases, of which 73 (52.1%) were male and 67 (47.9%) were female (*p*= 0.673).

### Association of CL/P and family history, pregnancy abnormalities, syndromes, and comorbidities

**Fig. 1:**
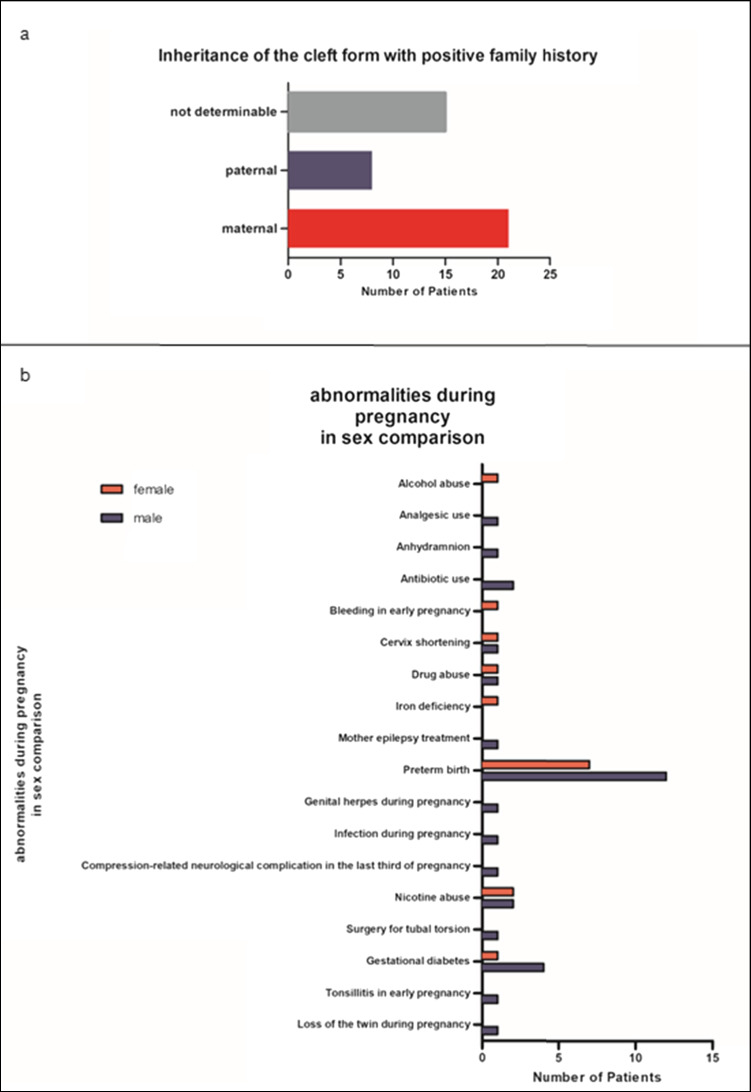
**a** Positive family history of orofacial clefts; **b** abnormalities during pregnancy

One out of nine patients (*n*=44, 10.9%) had a positive family history regarding CL/P (boys: *n*=28, 63.6%; girls: *n*=16, 36.4%; *p*=0.096; see Fig. 1 for a detailed description of the degree of the relationship and the number of affected relatives). The maternal to paternal inheritance ratio was 2.6:1.

Figure 1b displays the pregnancy abnormalities data, which occurred in 46 (11.4%) cases. Premature birth was the most frequent condition, with a total of 19 (41.3%) patients (boys: *n*=12, 63.2%; girls: *n*=7, 36.8%).

**Fig. 2:**
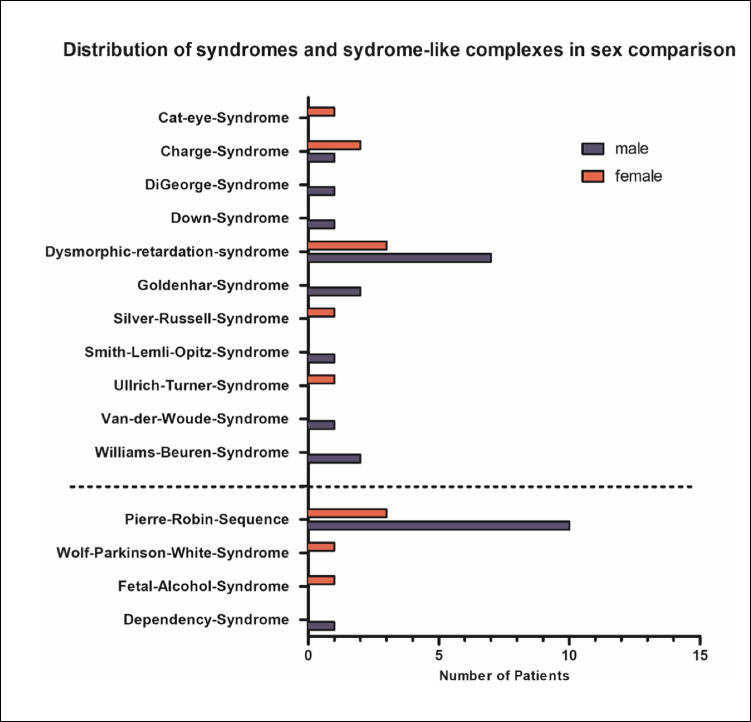
Distribution of syndromes and syndrome-like complexes according to sex: true syndromes are shown above the dashed line; syndrome-like complexes are shown below the dashed line

Furthermore, the patient population was evaluated regarding the occurrence of syndromic and non-syndromic clefts. Forty (9.9%) patients had syndromic clefts, while 354 (87.6%) patients showed non-syndromic clefts. In 10 cases, syndromic and non-syndromic clefts could not be precisely distinguished based on the records. Pierre Robin sequence was the most common syndrome-like observation, with 13 (32.5%) affected patients (see Fig. 2 for all observed syndromes and syndrome-like complexes). According to the Pierre Robin Consensus Conference 2016, it is now called a sequence and may be syndromic or non-syndromic. Due to the documentation, it was not possible to distinguish between syndromal and non-syndromal robin sequences so we grouped them all under syndromal-like complexes.

With 10 male and 3 female patients, the sex distribution for Pierre Robin sequence showed a ratio of 3.3:1.

A total of 122 (30.2%) patients in the study sample had at least one additional comorbidity (syndromic: *n*=29, 23.8%; non-syndromic: *n*=93, 76.2%; see Table 2 for detailed information).

**Table 2:** Cooccurring comorbidities in patients with a non-syndromic cleft according to cleft type (*n*=122)

*Comorbidity*	*CL*	*CLA*	*CLP*	*CP*	*Facial cleft*	*Total*
Heart and vessels	2	4	18	8	1	33
Speech development disorder	2	3	20	14		39
Ear/hearing		1	2	7	1	11
Genitourinary system	3		4			7
Extremities			4	3	1	8
Skeleton	1			4		5
Airway			2	6		8
Eyes	2		2	1	1	6
CNS			1	3		4
Neoplasia/tumor	2		2	1		5
Abdomen				1		1
Skin	1		3	1		5
Muscular system	1			1		2
Genome modification			1	2		3
Behavioral/emotional disorders	1		1	1		3
Blood			1			1
Facial malformation				3		3
Infectious diseases				1		1
Others	1		1	5		7

In total, 265 comorbidities occurred in the study sample. The number of comorbidities ranged from a single additional disease to as many as 10 other diseases. On average, a patient with a CL/P was affected by 2.17 cooccurring defects.

The association of cleft type and pregnancy abnormalities, the occurrence of syndromes and comorbidities is displayed in Table 3. While there was no significant association between the cleft type and pregnancy abnormalities, the presence of syndromes and comorbidities differed significantly among the cleft types. The presence of syndromes was most common in patients with a CP. Comorbidities were mostly associated with patients with a CLP and CP.

**Table 3:** Association of cleft type and pregnancy abnormalities, the occurrence of syndromes and comorbidities

	*CL* *n (%)*	*CLA* *n (%)*	*CLP* *n (%)*	*CP* *n (%)*	*Facial cleft*	*p value*
Pregnancy Abnormalities *n*=46 (100%)	6 (13%)	4 (8.7%)	15 (32.6%)	19 (41.3%)	2 (4.4%)	0.731
Syndromic cleft *n*=40 (100%)	1 (2.5%)	2 (5%)	9 (22.5%)	26 (65%)	2 (5%)	<0.001
Comorbidities *n=*122 (100%)	7 (5.7%)	7 (5.7%)	52 (42.6%)	51 (41.8%)	5 (4.1%)	0.001

### Need for surgery

In total, 931 cleft surgeries were performed during the analyzed period. As expected, patients with a CLP received the most surgeries (*n*=564; 60.6%), followed by patients with a CP (*n*=165; 17.7%), CLA (*n*=90; 9.7%), and CL (*n*=86; 9.2%). A total of 2.8% (*n*=26) of all surgeries were carried out for patients with a facial cleft. This means that on average, CL, CLA, CLP, and CP resulted in 1.5, 2.3, 3.6, and 1.2 surgeries per patient, respectively.

Of the 404 patients treated at our cleft center, 353 were treated according to the protocol described above. In the cohort of 353 patients, 6 patients (1.6%) needed lip revision 1 CLP patient needed a velopharyngoplasty and 2 patients needed a residual hole closure.

In 43 (10.6% of 404) patients who were initially treated elsewhere/outside of the protocol, 59 corrective surgeries were necessary. This included residual hole closures (*n*=36; 61%), velopharyngoplasties (*n*=22; 37%), and scar corrections (*n*=1, 1.6%). Patients with CLP or CP were more likely to receive corrective surgery than patients with CL (1 operation for a patient with a CL, 2 operations for a patient with a CLA); 28 operations were performed for patients with CLP and an additional 28 were performed for patients with a CP (*p*=0.001).

## Discussion

The present study provides the epidemiological and clinical data of patients with CL/P of a single center in lower saxony Germany investigated between 2001 and 2019. In total, the side could not be determined in 7 patients; however, these patients were still taken into account because they are meaningfully concerning the evaluation of the clinical profile apart from cleft side manifestation. With a prevalence of 1 patient with a CL/P in every 890 live births, the observed prevalence was slightly higher than the expected prevalence of 1 in every 1000 births for European countries. However, the data on prevalence differ widely and show geographic variations [2, 15]. In addition to the calculation of prevalence used here, there are other studies that provide extensive registry data, for example, in the Scandinavian countries. These are superior to the present survey but do not represent the same specific geographic area [22, 23].

The global trend of higher CL/P occurrence in boys was confirmed in the present study sample. Subgroup analysis revealed that this effect was caused by CLP, while CL, CLA, and CP were distributed equally between the sexes. In accordance with the existing literature, in unilateral cases, the left side was more often affected than the right side, and unilateral CL/P was more frequently observed than bilateral CL/P [10, 24–29].

In the study population, CLP occurred most frequently, followed by CP, CL, and CLA. This result is equal to the previously reported prevalence [10] but may also be affected by local factors, as exogenous and endogenous influences may vary for different geographic locations. In contrast, a Colombian study found CL to be the most common type of cleft [30]. Furthermore, genetic factors play a crucial role in CL/P occurrence, and a positive family history increases the risk of cleft manifestation [8, 31–34]. Compared to the international literature, the percentage of patients with a positive family history in the present study sample was rather low. However, a hereditary component in the development of cleft formation is undeniable even though the exact genetic mechanism is not yet fully understood, and more than 50 genes have been described to be associated with non-syndromic patients with a CL/P [35]. For instance, *T-box* and *MSX* genes are believed to play a major role in palatogenesis[36–38]. Interestingly, these gene families are also involved in heart development [39–42]. Therefore, genetics might explain the high co-occurrence of CL/P and heart disease observed in the presented sample. However, further contributing factors must be assumed. For example, stressful life events during pregnancy may be associated with higher risk of CL/P and congenital heart diseases [43–45]. The maternal stress activates the hypothalamus-pituitary-adrenal axis, increases glucocorticoid production, and might cause gene-environment interactions. Mostaka et al. identified the stress-related genes SLC6A4, TPH2, and SERPINA6 to increase the risk of having a child with orofacial cleft [46].

We observed inheritance primarily from the maternal side, which could lead to the assumption that clefts are caused by the maternal genotype working through the prenatal environment. However, this has been disproved, and there is some evidence that the fetal genes themselves make the major genetic contribution to CL/P [47]. Moreover, other epidemiological studies showed no difference in family recurrence between affected mothers and fathers [48] or a pronounced hereditary component from the paternal side [33, 49, 50].

Furthermore, blood relationship between parents is seen as a risk factor for the development of CL/P. Kin marriages, which are practiced in some cultures, underline the correlation of cultural and genetic influences on CL/P prevalence [51]. However, as genetics, genomics, and epigenetics contribute to cleft formation, the etiology of CL/P is too complex to be explained by inheritance only. Epigenetic-wide association studies provided evidence that DNA methylation, histone modifications, and non-coding RNA might explain the missing heritability For multifactorial inheritance, it is assumed that the risk of transmission to subsequent generations decreases as the degree of relatedness decreases [52]. The distribution of the degrees of relatedness in our patient cohort supports this hypothesis.

Preterm birth was the most common abnormality during pregnancy in our study population, and a higher prevalence of preterm births in mothers of patients with a cleft was observed previously [8, 10]. Shehan et al. (2021) reported a 1.9 times higher likelihood of being born with CL/P for preterm infants than for full-term infants [53]. This correlation is not surprising, as there are several risk factors that contribute to both CL/P and prematurity, such as maternal smoking or substance abuse. However, an interactive pathogenesis behind this correlation has not been described [53]. Therefore, prematurity and CL/P are probably caused independently from each other.

A similar approach applies for the association of CL/P and syndromes or. This is reflected by the fact that the percentage of abnormalities during pregnancy in syndromic patients with CL/P was twice that in patients with non-syndromic clefts.

In the present study population, syndromic CL/P accounted for 10.4% of all orofacial clefts and thus falls into the wide range of reported percentages of syndromic cleft formation in the literature, which varies from 4.8% to almost 30% [10, 54, 55].

At least 487 syndromes may be associated with cleft formation [56]. In Goettingen, 15 different syndromes and syndrome-like complexes were observed during the study period.

The Robin sequence was found most frequently, in accordance with the literature [56–58].

One-third of our patients displayed at least one comorbidity with a higher probability when the palate was also affected by the cleft. The most common comorbidities were heart diseases followed by respiratory tract diseases and language development disorders. These findings were similar to other epidemiological studies [8, 10, 54, 59] and support the need for the comprehensive pediatric assessment of infants with a CL/P, especially with regard to congenital heart diseases, which occur in 3.9 to 23.9% of all patients with a CL/P. It must be mentioned here that no distinction was made between syndromic and non-syndromic Robin sequences and a direct conclusion from the sequence to an expected comorbidity, which could be explained by a syndrome is difficult. The observation that patients with palate involvement are at higher risk for other organ abnormalities and comorbidities suggests that a relationship between the timing of cleft appearance and genes responsible for organ development requires further exploration to better understand the development of clefts [60].

In addition to the treatment strategy, the skills and experience of the surgeon is essential for a satisfactory rehabilitation of patients with CL/P. There are 194 different concepts at 201 cleft centers in Europe, which underscores the importance of tradition at the respective center [21]. Consequently, several surgical techniques for lip and palatal closure have been postulated. In our center, surgical closure of the lip is performed using the Tennison and Randall technique supplemented by simultaneous plastic surgery according to Axhausen in the case of ridge involvement [61].

For palate closure, our center differentiates between isolated CP and CLP. In the case of CP only, the cleft palate is repaired in one-step with a pedicle flap plasty according to Veau [62]. In the case of CLP, palate closure is a two-step process after lip repair. First, Pichler plasty is performed to close the hard palate [63]. After 3 months, analogous to CP only, the soft palate is closed using the pedicle flap-plasty according to Veau.

The rate of corrective surgery in the present study was 10.6%, and regarding the subcollective of patients who received primary surgery according to the University Medical Center Goettingen treatment protocol, the rate was 2.2% and therefore lower compared to other studies, which reported corrective surgeries in 33%, 56.9%, and 69.3% of all treated cases [64–66].This low rate can reflect the center’s restrictive approach regarding corrective surgery and might provide an argument for a stringent treatment protocol. However, surgical and clinical experience plays a major role in cleft surgery, not only the capacity of the surgeon itself but of the whole team can improve the outcome [67, 68].

There are some limitations to this study, including that the data were acquired retrospectively. The analysis includes all patients presented to our center since the introduction of the treatment concept. Consequently, all age groups were represented in the patient collective. Due to the concept, the primary operations of the cleft for complete closure were all completed within the first 15 months of life. Certainly, this is a patient population in flux, as patients who presented as newborns in 2019 had not completed 18 years of life by the time data collection was completed, and further surgical interventions were pending in accordance with the treatment plan. Thus, interdisciplinary care continues through the consultation for all patients who are not yet adults. Furthermore, medical records can contain errors and be incomplete. Even though most of the documentation was accomplished by the same surgeon, some records were completed by his representative. Moreover, the results are very specific for the area of Lower Saxony in Germany and may not be generalizable to other populations. However, this focus was chosen on purpose because data for this geographic area were missing.

In summary, the epidemiological and clinical characteristics of patients with cleft in Lower Saxony Germany are consistent with the literature. The large number of comorbidities calls for an interdisciplinary approach with special attention to the potential presence of cardiovascular disease in non-syndromic patients with a CL/P. A stringent treatment protocol and multidisciplinary approach is essential in oro-facial cleft care.

## Data Availability

The data that support the findings of this study are available from the corresponding author K.P., upon reasonable request.
